# Supplementary material for: Monitoring of drought stress and transpiration rate using proximal thermal and hyperspectral imaging in an indoor automated plant phenotyping platform
Source: Plant Methods. 2023 Nov 23;19:132. doi: 10.1186/s13007-023-01102-1 (PMC10668392; doi:10.1186/s13007-023-01102-1)
Supplement: Supplementary file 2 — Additional file 2: Figure S1. Baselines of the crop water stress indices. This figure illustrates the baselines used to calculate the Idso crop water stress index (ICWSI), development-corrected crop water stress index (CWSIdev) and air temperature (Ta) corrected crop water stress index (CWSITa) inside the imaging cabin. Similar baselines were created for the other monitoring positions (outside the cabin, growth zone). In A and B, the baselines of young and mature plants are represented by a black and yellow line, respectively, while the individual measurements are indicated with black and yellow dots. A baselines of the ICWSIin index, which relates plant temperature (Tp) to vapor pressure deficit (VPD). Separate baselines were created for the different genotypes and young/mature plants. This baseline was used to estimate plant temperature (Tp) of a fully transpiring plant. B the baselines of the CWSIdev_in, which relates canopy temperature depression (CTD = Tp-Ta) to VPD. This function is used to estimate the CTD of a fully transpiring plant. Separate baselines for genotypes and developmental stages were also created for this index. C, representation of the baselines used to calculate the CWSITa_in. The model of this baseline has CTD as the dependent variable and VPD, Ta and its interaction term as independent continuous variables. This figure illustrates what the relationship between CTD and VPD would look like if Ta was constant. The relationships between CTD and VPD for eight different temperatures are indicated by solid lines. The measurements are visualized by slightly transparent dots. Each temperature has received a unique color, which is used for both the line and dots. Separate baselines were developed for each genotype. Figure S2. Environmental data. The daily mean air temperature (Ta, A), relative humidity (RH, B) and vapor pressure deficit (VPD, C) of the three monitoring positions (gz, in and out) are represented by a solid light gray line, dashed dark [file 13007_2023_1102_MOESM2_ESM.pdf]

Supplementary Information

Monitoring of drought stress and transpiration rate using proximal thermal infrared and hyperspectral imaging in an indoor automated plant phenotyping platform

Stien Mertens, Lennart Verbraeken, Heike Sprenger, Sam De Meyer, Kirin Demuynck, Bernard Cannoot, Julie Merchie, Jolien De Block, Jonathan T. Vogel, Wesley Bruce, Hilde Nelissen, Steven Maere, Dirk Inzé* and Nathalie Wuyts

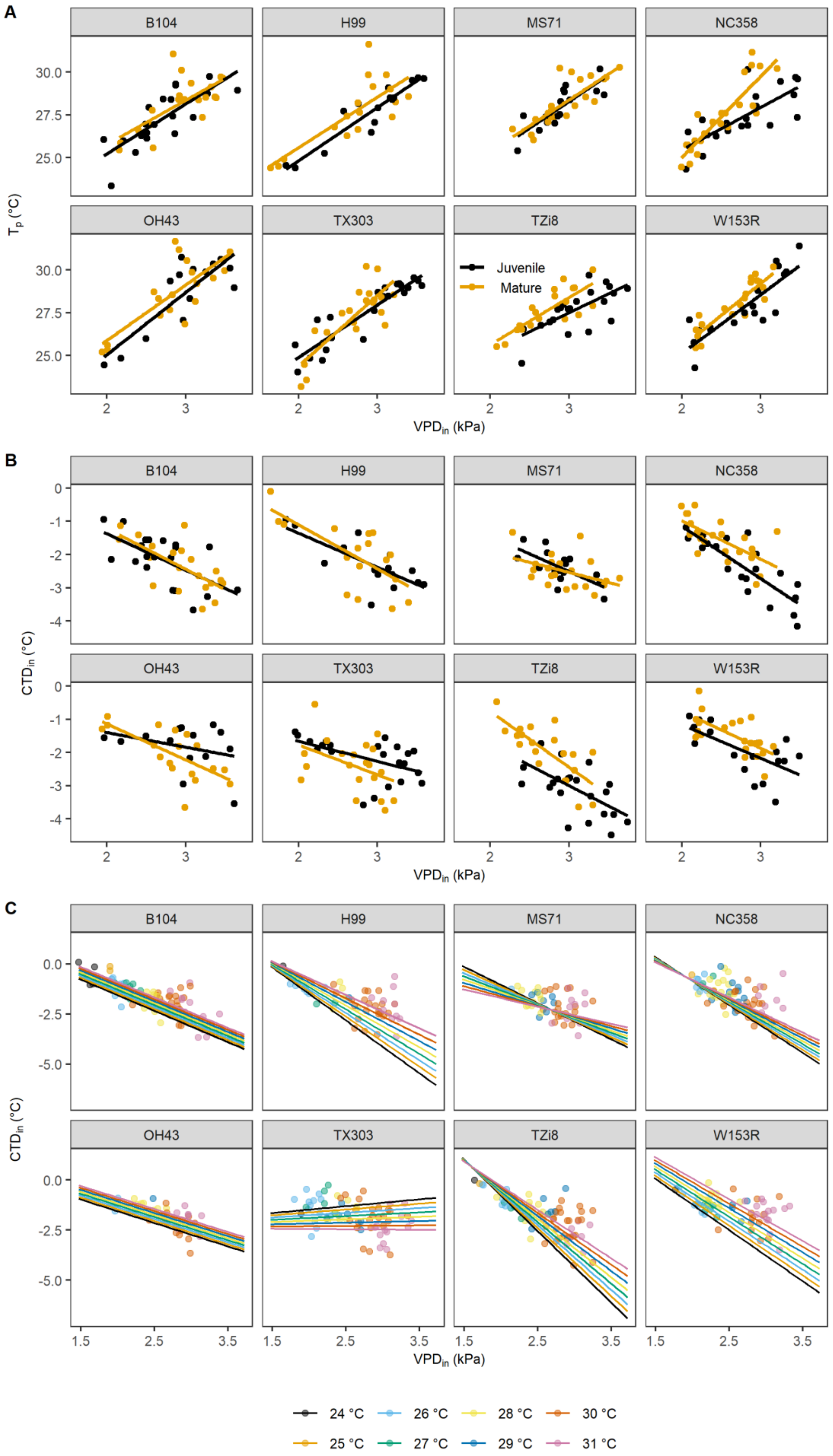

A
B104
H99
MS71
NC358
OH43
TX303
TZi8
W153R
Juvenile
Mature
Tp (°C)
VPDin (kPa)
B
CTDin (°C)
C
24 °C
26 °C
28 °C
30 °C
25 °C
27 °C
29 °C
31 °C

Figure S1. Baselines of the crop water stress indices. This figure illustrates the baselines used to calculate the Idso crop water stress index (ICWSI), development-corrected crop water stress index ($CWSI_{dev}$) and air temperature (T_a) corrected crop water stress index ($CWSI_{Ta}$) inside the imaging cabin. Similar baselines were created for the other monitoring positions (outside the cabin, growth zone). In A and B, the baselines of young and mature plants are represented by a black and yellow line, respectively, while the individual measurements are indicated with black and yellow dots. **A**, baselines of the $ICWSI_{in}$ index, which relates plant temperature (T_p) to vapor pressure deficit (VPD). Separate baselines were created for the different genotypes and young/mature plants. This baseline was used to estimate plant temperature (T_p) of a fully transpiring plant. **B**, the baselines of the $CWSI_{dev\_in}$, which relates canopy temperature depression (CTD = T_p-T_a) to VPD. This function is used to estimate the CTD of a fully transpiring plant. Separate baselines for genotypes and developmental stages were also created for this index. **C**, representation of the baselines used to calculate the $CWSI_{Ta\_in}$. The model of this baseline has CTD as the dependent variable and VPD, T_a and its interaction term as independent continuous variables. This figure illustrates what the relationship between CTD and VPD would look like if T_a was constant. The relationships between CTD and VPD for eight different temperatures are indicated by solid lines. The measurements are visualized by slightly transparent dots. Each temperature has received a unique color, which is used for both the line and dots. Separate baselines were developed for each genotype.

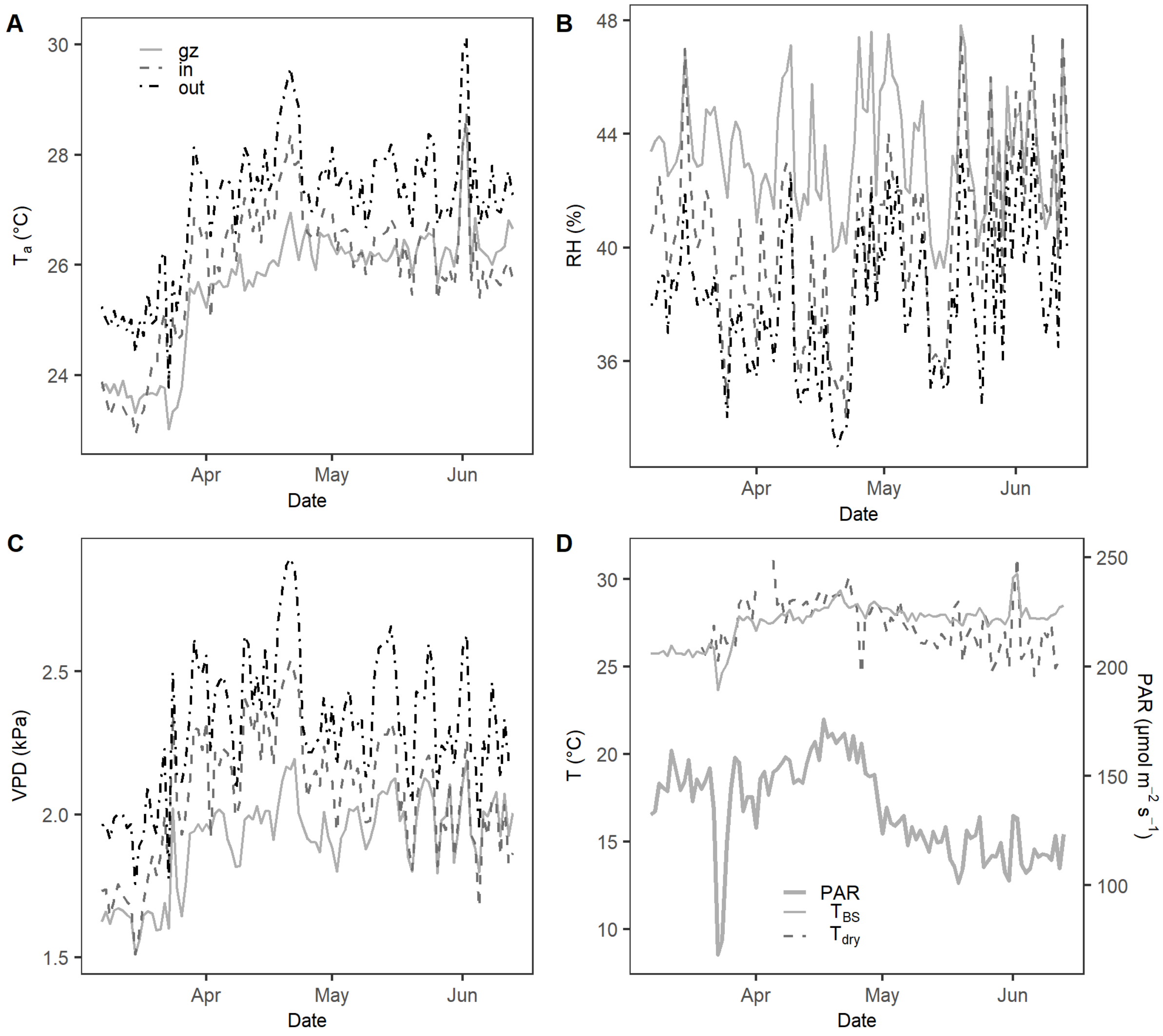

Figure S2. Environmental data. The daily mean air temperature (T_a, **A**), relative humidity (RH, **B**) and vapor pressure deficit (VPD, **C**) of the three monitoring positions (gz, in and out) are represented by a solid light gray line, dashed dark gray line and dot-dashed black line, respectively. **D** shows the measurements of the black sphere temperature (T_{BS}), PAR monitored in the growth zone, and the dry reference temperature (T_{dry}) that was measured inside the imaging cabin. The daily mean of T_{BS} and PAR are indicated by a thin and thick light gray line, respectively, while T_{dry} is represented by a dashed dark gray line.

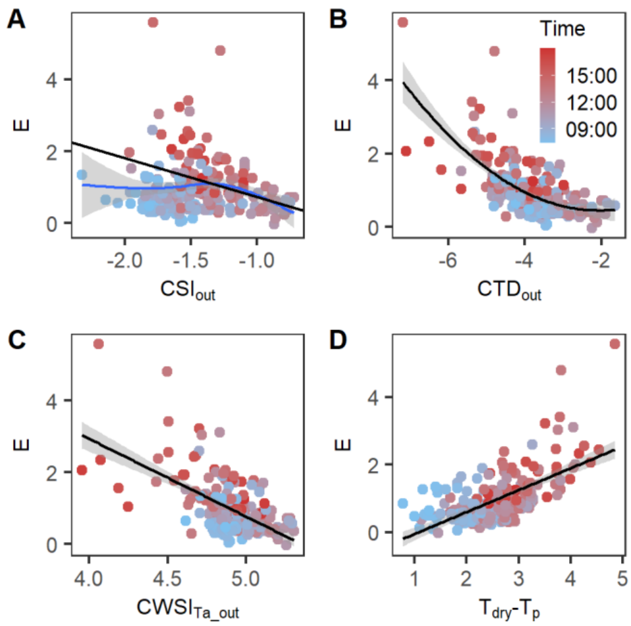

Figure S3. Relationship between thermal infrared indices and transpiration rate. Individual measurements are represented with colored dots showing the VPD_{out} at the time of sampling. A blue-red gradient is used to visualize the time range. The linear or polynomial relationships between the indices are indicated with a black line, while non-linear (spline) relationships are represented by a blue line. The gray shading around the lines show the 95% confidence interval of the relationship. Transpiration rate (E, mmol $m^{-2}s^{-1}$) versus (**A**) CSI_{out}, (**B**) CTD_{out}, (**C**) $CWSI_{Ta\_out}$, and (**D**) T_{dry} -T_p (°C).

Table S1. Temperature and photosynthetically active radiation ranges in the growth zone.

Experiment	Time	T_a	PAR
TF	10:00	27.1±1.8 - 27.8±1.8	177.2±74.1 - 225.2±103.7
	13:00	27.7±1.8 - 28.2±2.0	202.5±76.2 - 289±187.4
	15:00	28.7±2.4 - 29.1±2.5	176.1±66.0 - 252.7±133.0
DR	10:00	28.1±0.4 - 29.2±0.6	203.6±88.1 - 230.1±98.7
	13:00	27.7±0.4 - 28.2±0.4	136.4±72.4 - 158.5±72.2
	15:00	29.3±0.5 - 30.1±0.7	127.3±82.8 - 155.9±83.8

The ranges are calculated by averaging temperature (T_a, °C) and photosynthetically active radiation (PAR, µmol $m^{-2}s^{-1}$) measurements for each weather station. The table provides the average temperature and PAR ranges (including standard deviations) at 10.00, 13.00 and 15.00 for each experiment.

Table S2. Measurement details including frequency, time of day, developmental stage and duration, for imaging and physiological measurements in the drought and transferability experiment.

Exp	Measurement	Frequency	Time	n	Start	End	Duration
DR	Imaging	Daily	7.30-14.00	25	V1	V11	44
	Gas exchange	Weekly	7.30-14.00	25	V3	V11	44
TF	Imaging	Daily	7.30-14.00	306	V1	silking	99
		Punctual:	13.00-18.00	144			
		1) V5+10 days			V5-V8*	na	na
		2) V13±2 days			V13-V15*	na	na
	Gas exchange, leaf water potential and fluorescence	Weekly	7.30-14.00	48	V3*	V23*	99
		Punctual:	13.00-18.00	144			
		1) V5+10 days			V5-V8*	na	na
		2) V13±2 days			V13-V15*	na	na

Exp: experiment; DR: drought experiment; TF: transferability experiment; n: number of plants; Start and End: developmental stage at which the first and last measurement, respectively, took place, expressed in V stage; Duration: duration of the experiment expressed in number of days; na: not applicable; *: depending on the genotype, see Table S3.

Table S3. Developmental stages expressed in V stages at which physiological measurements were performed for the different genotypes in the drought and transferability experiment.

Experiment	Frequency	B104	H99	MS71	NC358	OH43	TX303	Tzi8	W153R
DR	Weekly	3-11	na	na	na	na	na	na	na
TF	Weekly	4-21	3-16	4-18	5-22	4-14	4-20	4-23	3-15
	Punctual:								
	V5+10d	6-8	6-8	6-8	6-8	6-8	5-8	6-7	5-7
	V13±2d	13-14	14-15	13-15	13-14	14-15	13-14	13-14	13-15

DR: drought experiment; TF: transferability experiment; na: not applicable.
